# Supplementary material for: LDLR is used as a cell entry receptor by multiple alphaviruses
Source: Nat Commun. 2024 Jan 20;15:622. doi: 10.1038/s41467-024-44872-5 (PMC10799924; doi:10.1038/s41467-024-44872-5)
Supplement: Supplementary file 1 — Supplementary Information [file 41467_2024_44872_MOESM1_ESM.pdf]

Supplementary Information

LDLR is used as a cell entry receptor by multiple alphaviruses

Xiaofeng Zhai[1#], Xiaoling Li[1#], Michael Veit[2#], Ningning Wang[1], Yu Wang[1], Andres Merits[3], Zhiwen Jiang[1], Yan Qin[1], Xiaoguang Zhang[1], Kaili Qi[1], Houqi Jiao[1], Wan-Ting He[1], Ye Chen[4], Yang Mao[5]*, Shuo Su[1]*

[1] Engineering Laboratory of Animal Immunity of Jiangsu Province, College of Veterinary Medicine, Academy for Advanced Interdisciplinary Studies, Nanjing Agricultural University, Nanjing, China.

[2] Institute for Virology, Center for Infection Medicine, Veterinary Faculty, Free University Berlin, Berlin, Germany.

[3] Institute of Technology, University of Tartu, Nooruse Street 1, 50411, Tartu, Estonia.

[4] Key Laboratory of animal pathogen infection and Immunology of Fujian Province, College of Animal Sciences, Fujian Agriculture and Forestry University, Fuzhou 350002, China.

[5] School of Pharmaceutical Sciences and National-Local Joint Engineering Laboratory of Druggability and New Drugs Evaluation, Sun Yat-sen University, Guangzhou, China.

*Corresponding author.

[#]Contributed equally.

E-mail: Shuo Su (shuosu@njau.edu.cn); Yang Mao (maoyang3@mail.sysu.edu.cn);

Supplementary Fig. 1

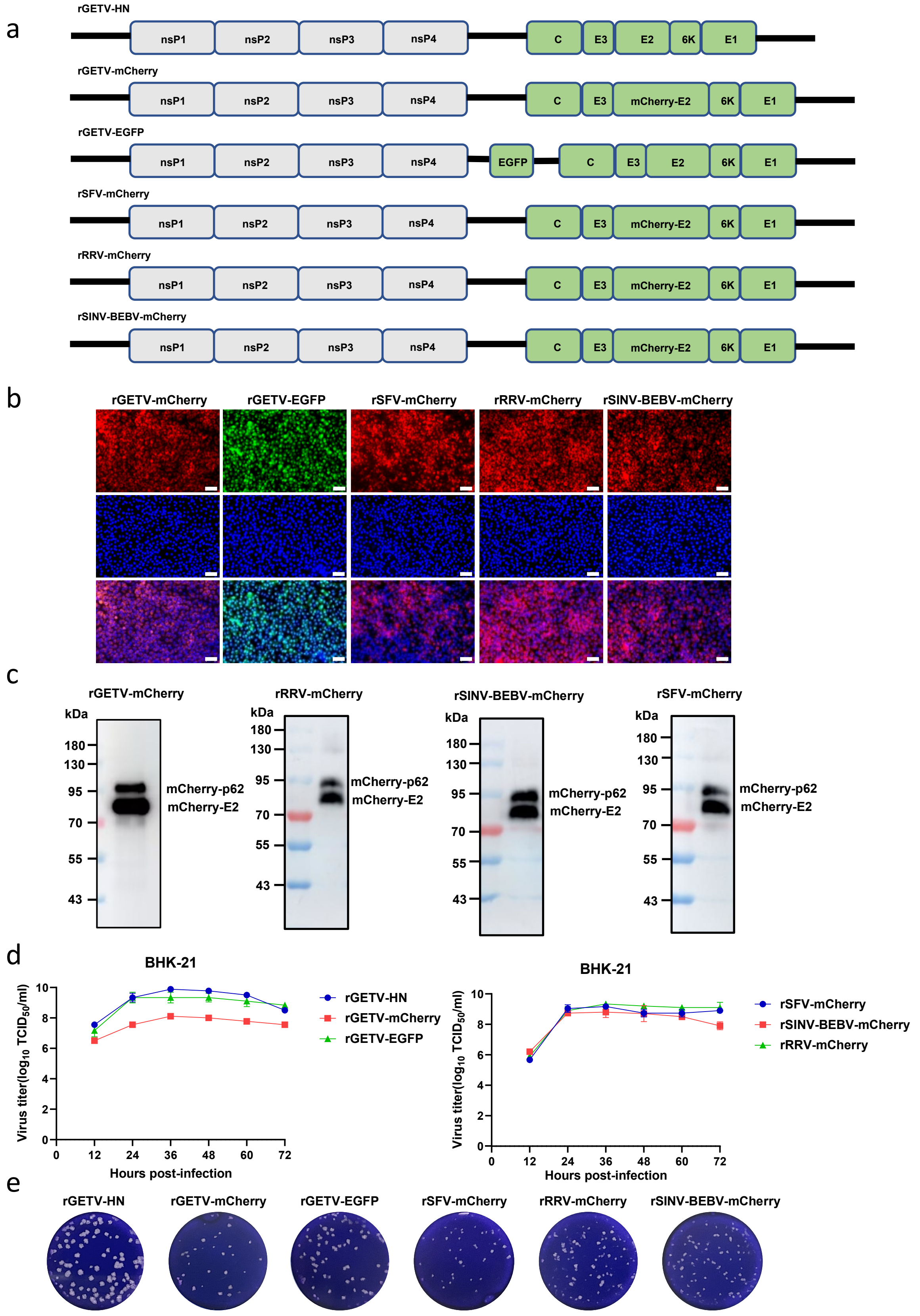

Supplementary Fig. 1 Generation of replication-competent alphaviruses harboring reporter genes. a, Schematic illustration of the genomes of rGETV-HN, rGETV-mCherry, rGETV-EGFP, rSFV-mCherry, rRRV-mCherry and rSINV-BEBV-mCherry. **b**, Expression of fluorescent proteins in BHK-21 cells infected with the rGETV-mCherry, rGETV-EGFP, rSFV-mCherry, rRRV-mCherry and rSINV-BEBV-mCherry. Nuclei were counterstained with DAPI. Scale bar, 250 μm. **c**, Expression of mCherry-E2 and mCherry-p62 fusion proteins in rGETV-mCherry, rRRV-mCherry, rSINV-BEBV-mCherry, and rSFV-mCherry infected cells was analyzed by western blot. Bands corresponding to fusion proteins were detected using anti-mCherry antibody. **d**, One-step growth curves of the rGETV-HN, rGETV-mCherry, rGETV-EGFP (left panel), rSFV-mCherry, rRRV-mCherry and rSINV-BEBV-mCherry (right panel). BHK-21 cells were infected with different viruses at an MOI of 0.01. Titers are shown in $TCID_{50}$ per ml. Mean ± SD of 3 independent experiments are shown. **e**, The plaques of the rGETV-HN, rGETV-mCherry, rGETV-EGFP, rSFV-mCherry, rRRV-mCherry and rSINV-BEBV-mCherry on BHK-21 cells. The experiment was performed twice with similar results. Molecular masses are indicated in kDa (**c**). Source data are provided as a Source Data file.

Supplementary Fig. 2

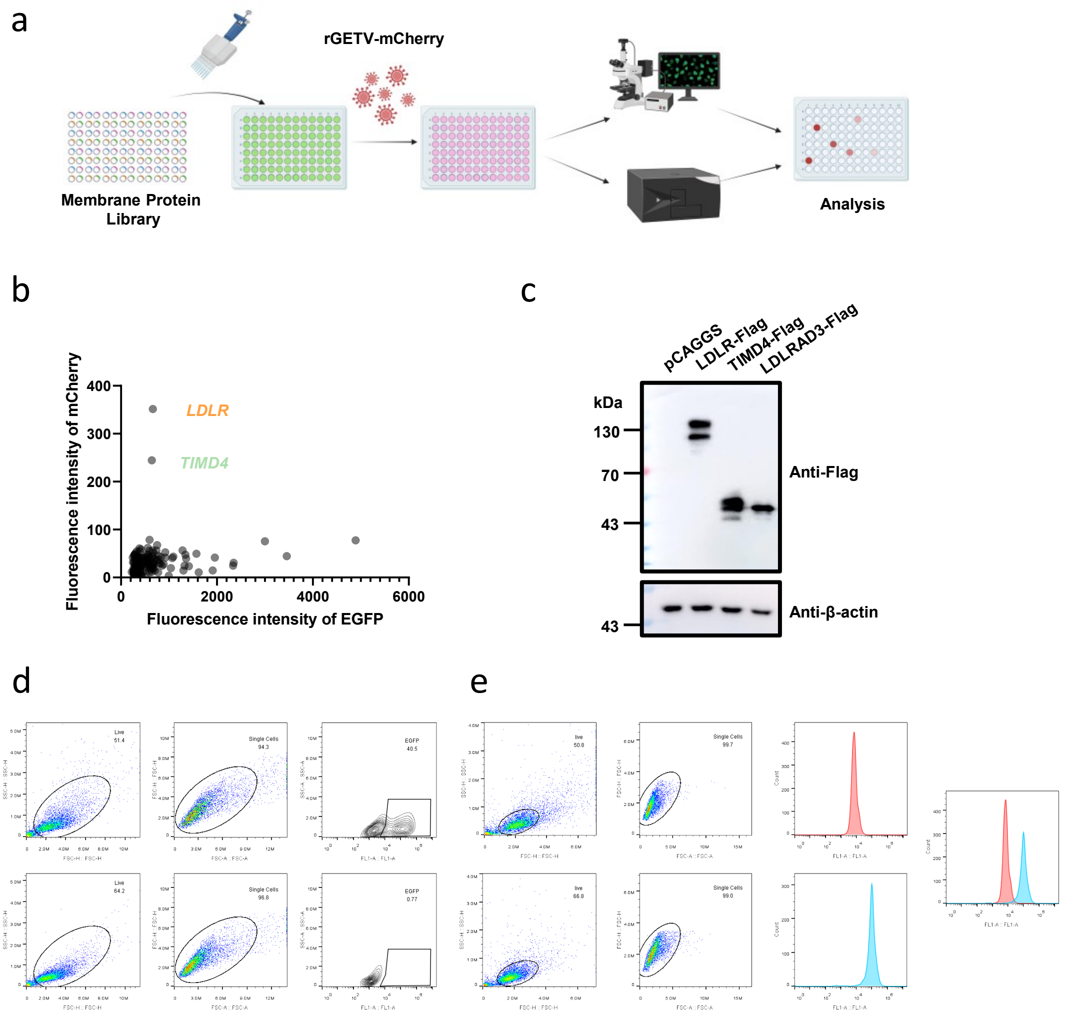

Supplementary Fig. 2 Cell receptor screen identifies LDLR as a factor boosting infectivity of GETV. a, Scheme of the overexpression-based screen. Plasmids encoding a small library of mouse membrane proteins and EGFP reporter were transfected into HEK 293T cells that were subsequently infected with rGETV-mCherry. The rGETV-mCherry infection (red fluorescence) and co-expressed of EGFP with membrane proteins (green fluorescence) were imaged by an inverted fluorescence microscope; fluorescence intensities were measured using an automatic microplate reader. Figures were generated with BioRender software (https://biorender.com/) under the terms of a site license. **b**, Overall results of the overexpression library screen. The y-axis represents the red fluorescence intensity resulting from rGETV-mCherry infection. The x-axis shows the green fluorescence intensity from the expression of mouse membrane proteins. Each circle represents a single protein. The fluorescence intensity of mCherry or EGFP were measured at the excitation 587 nm and 395 nm respectively, and normalized to those of untransfected cells. **c**, Western blot analysis of LDLR, LDLRAD3 and TIMD4 expression (all flag-tagged) in HEK 293T cells. Anti-Flag antibody was used as the primary antibody for detection. β-actin was used as loading control. **d**, Examples showing flow cytometry gating strategy for the quantification of EGFP expressing cells upon rGETV-EGFP infection. HEK 293T control cells (bottom panels) and cells expressing LDLR (top panels) were infected with rGETV-EGFP. The percentage of cells falling within each gate is shown in the upper right corner. **e**, Flow cytometry gating strategy for receptor detection after immunostaining. HEK 293T control cells (top panels) and cells overexpressing LDLR (bottom panels) were stained with anti-FLAG primary antibody and an Alexa Fluor 488 conjugated goat anti-mouse IgG as a secondary antibody. Results from flow cytometry analysis were overlaid to show positive receptor expression (the example is from an experiment in Supplementary Fig. 4a). All the experiments were performed at least twice.

Supplementary Fig. 3

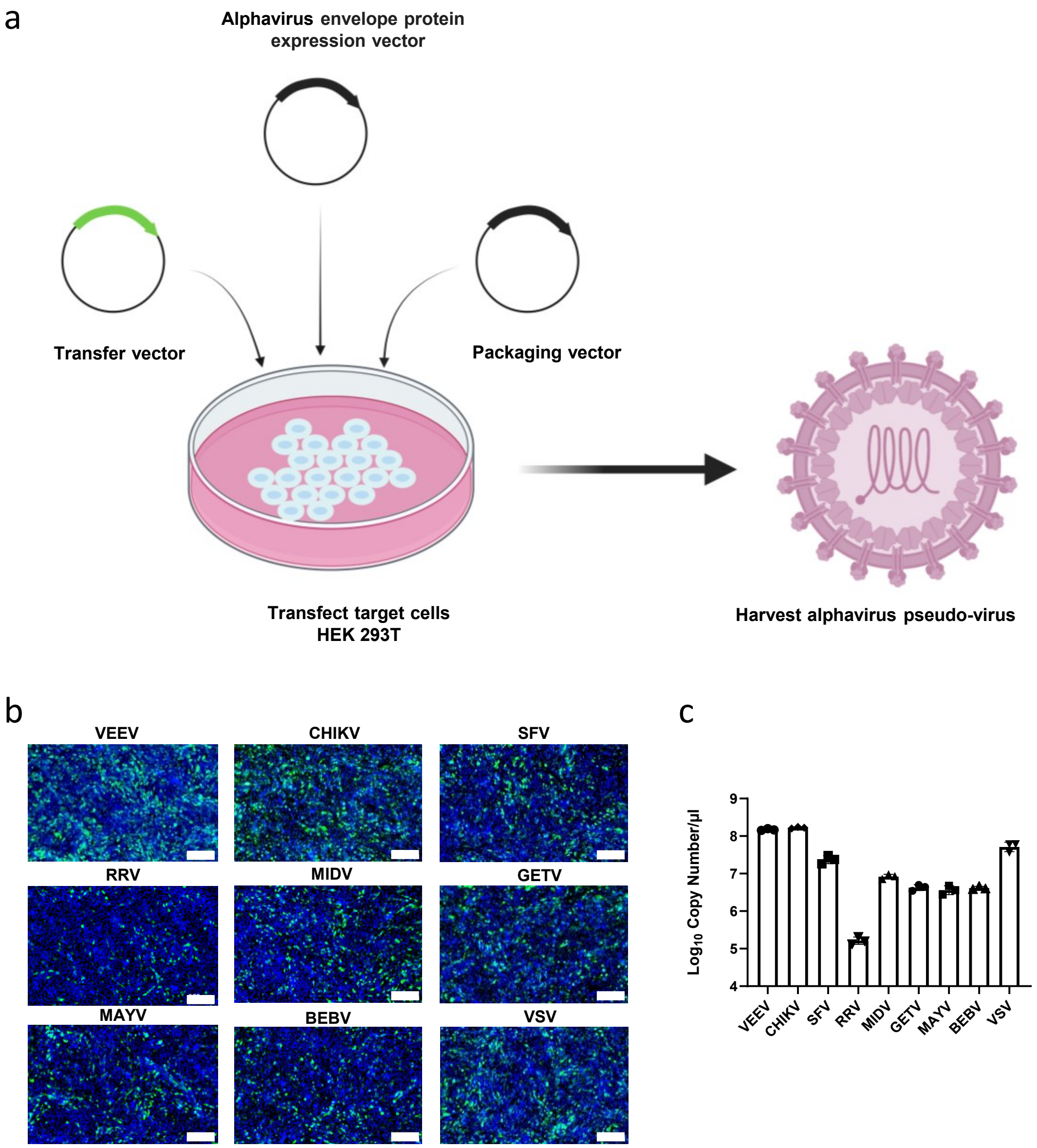

Supplementary Fig. 3 Construction of lentivirus particles pseudotyped with glycoproteins of alphaviruses. a, The scheme of pseudo-virus production. Region encoding for EGFP is shown with green arrow. Figures were created with BioRender software (https://biorender.com/) under the terms of a site license. **b**, Expression of EGFP in BHK-21 cells infected with pseudo-viruses harboring envelop proteins of viruses indicated above the panels. Cells were examined for the EGFP signal at 48 h post infection. Particles pseudotyped with G protein of VSV were used as control. Scale bar: 500 µm. **c**, Titers of obtained pseudo-viruses measured by qRT-PCR analysis. Primers detecting sequence encoding gag protein in the packaged lentivirus genomic RNA were used. Mean ± SD of three independent experiments are shown. Source data are provided as a Source Data file.

Supplementary Fig. 4

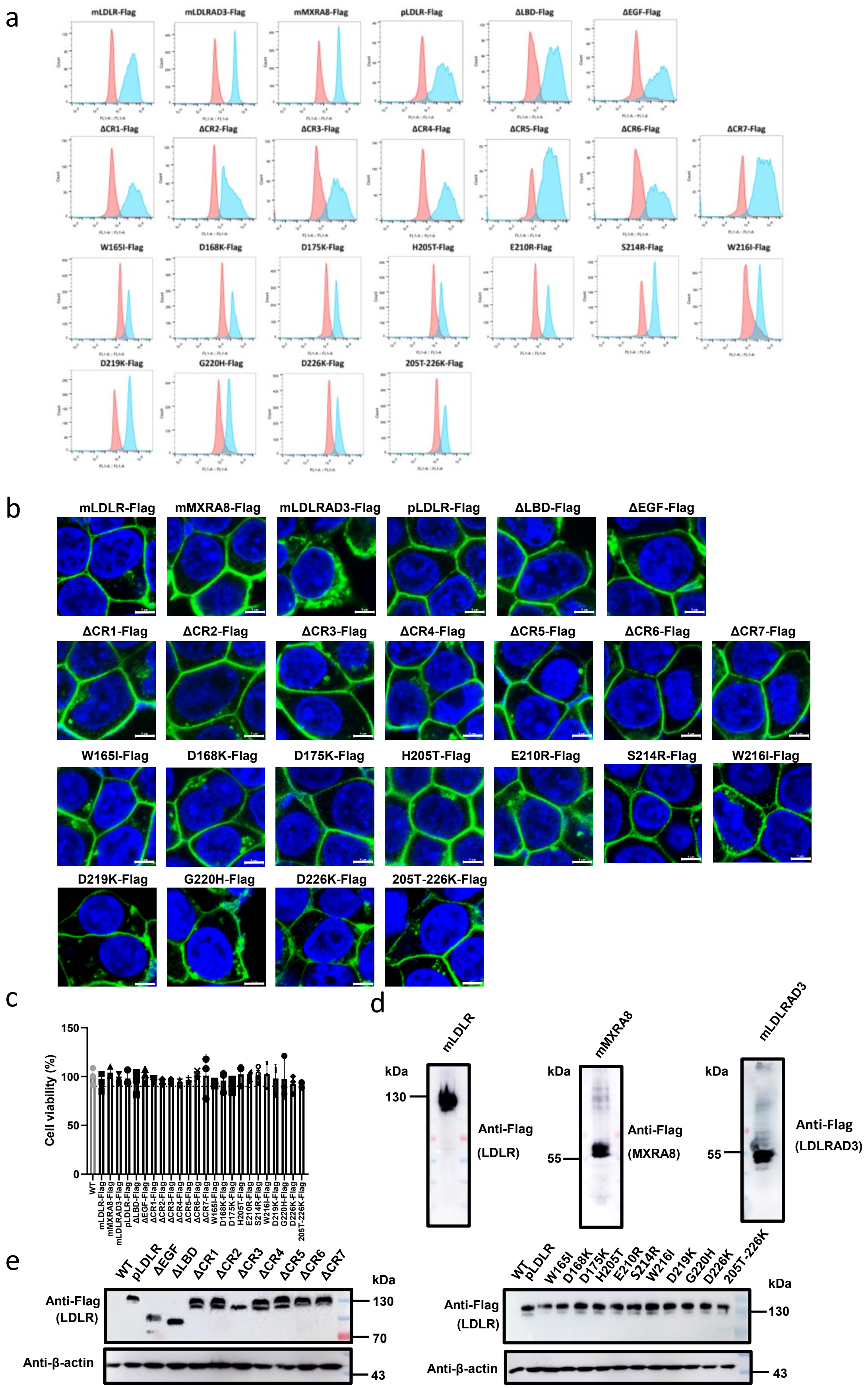

Supplementary Fig. 4 Analysis of the expression of cell surface receptors. **a**, Flow cytometry analysis of HEK 293T cells stably expressing indicated Flag-tagged proteins. Cells were stained with monoclonal anti-Flag M2 antibody (blue); isotype mouse IgG (pink) was used as a control. **b**, The localization of indicated Flag-tagged proteins in corresponding HEK 293T cell lines was detected by IFA. Proteins were detected using monoclonal anti-Flag M2 antibody (green), nuclei were counterstained with DAPI (blue). **c**, Viability of HEK 293T cell lines stably expressing indicated proteins was measured using Cell Counting Kit 8. Obtained values were normalized to these measured for parental HEK 293T cells (taken as 100%). Mean ± SD of three independent experiments are shown. **d-e**, HEK 293T cells stably expressing the indicated Flag-tagged proteins were lysed and presence of recombinant proteins was analyzed by western blot using antibody against the Flag-tag; antibody against β-actin was used to detect a loading control. The experiment was performed twice with similar results. Molecular masses are indicated in kDa (**d-e**). Source data are provided as a Source Data file.

Supplementary Fig. 5

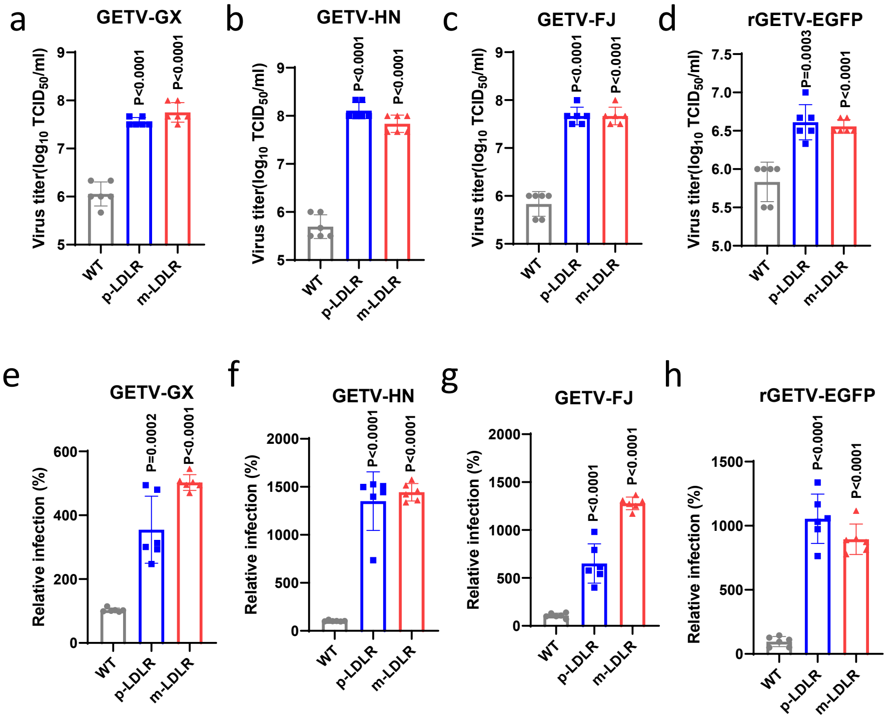

Supplementary Fig. 5 Analysis of GETV infection in cells overexpressing LDLR from pig or hamster. **a-h**, HEK 293T cells stably overexpressing LDLR from pig (p-LDLR) or hamster (m-LDLR) were infected with GETV-GX, GETV-HN, GETV-FJ or rGETV-EGFP (MOI=1). Virus titers in supernatants collected at 18 h post infection were determined on BHK-21 cells. WT: parental HEK 293T cells not expressing any exogenous LDLR (**a-d**). Infected cells were stained with anti-E2 mAb; the numbers of infected cells were determined by flow cytometry(**e-h**). Results are normalized relative to WT cells (e-h). Data are presented as mean values ± SD (n= 3 independent experiments) and two-tailed p-values are calculated by unpaired Student's t test. Source data are provided as a Source Data file.

Supplementary Fig. 6

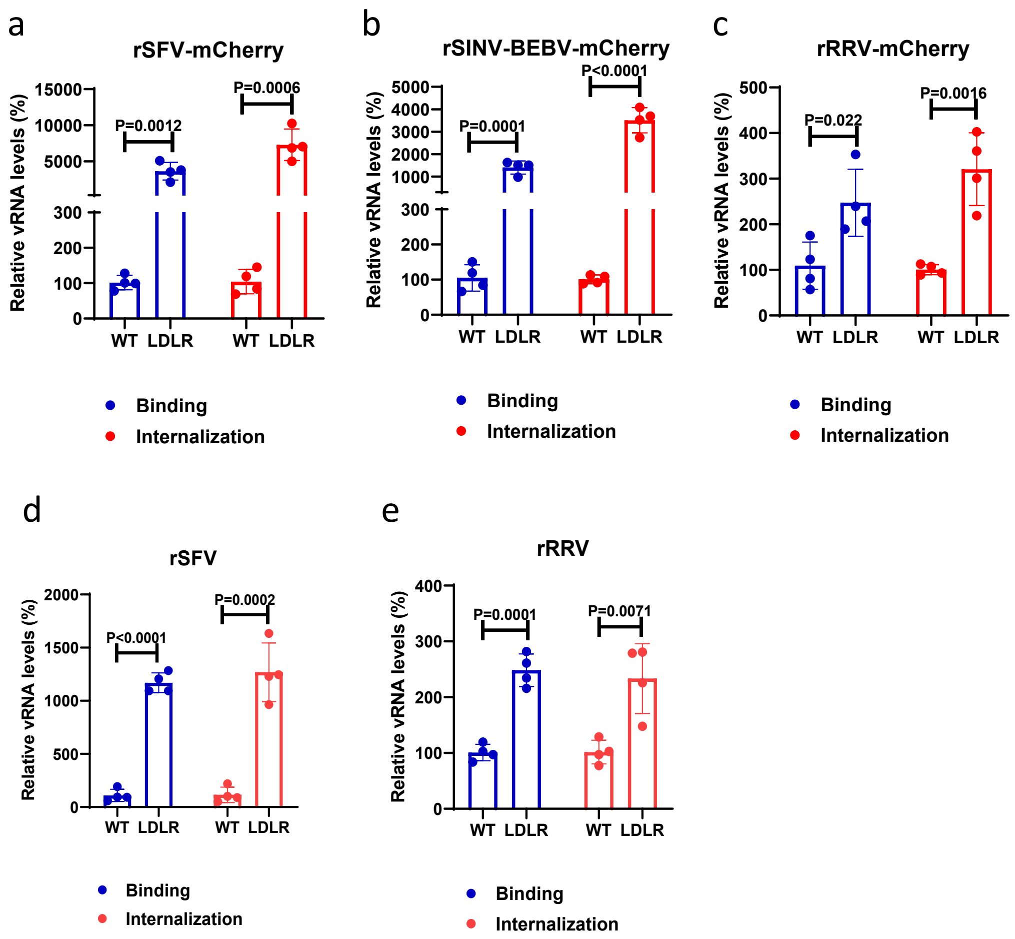

Supplementary Fig. 6 Expression of LDLR promote the entry of the virus. a-e, HEK 293T cells stably overexpressing LDLR from hamster were incubated with virions of rSFV-mCherry (**a**), rSINV-BEBV-mCherry (**b**), rRRV-mCherry (**c**), rSFV(**d**), or rRRV(**e**) at 4 °C for 30 min and harvested thereafter. Alternatively, after removing the unbound virus, cells were incubated further at 37° C for 1 h and harvested thereafter. vRNA levels were measured via qRT-PCR; data was normalized relative to WT cells. Mean ± SD of two independent experiments. Two-tailed p-values are calculated by unpaired Student's t test. Source data are provided as a Source Data file.

Supplementary Fig. 7

a

LLC-PK1 Δ*ldlr-1* cells
CCTGTGATGGTGATCCTGACTGCGAGGACGGCTCAGACGA wild-type allele
CCTGTGATGGTGATCCTG- - - - CGAGGACGGCTCAGACGA mutant allele
CCTGTGATGGTGATCCTGA- TGCGAGGACGGCTCAGACGA mutant allele
LLC-PK1 Δ*ldlr-2* cells
AGCCACAGCTCATCACTCCCCGAGAGGAGCAACAA wild-type allele
AGC- A- AG- AA mutant allele
AGCCACAGCTCATCAC- - CCCGAGAGGAGCAACAA mutant allele
ST Δ*ldlr-1* cells
CCTGTGATGGTGATCCTGACT- GCGAGGACGGCTCAGACGA wild-type allele
CCTGTGATGGTGATCCTGACTTGCGAGGACGGCTCAGACGA mutant allele
CCTGTGATGGTGATCCTGACT- - CGAGGACGGTTCAGACGA mutant allele
ST Δ*ldlr-2* cells
GCAGGAGCCACAGCTCATCACTCCCC- GAGAGGAGCAACAA wild-type allele
GCAGGAGCCACAGCTCATCACTCCCCCGAGAGGAGCAGCAA mutant allele
GCAGGAGCCACAGCTCATCACTCCCC- - AGAGGAGCAACAA mutant allele
BHK-21 Δ*ldlr-1* cells
AAGACGTGCTCCCCGGACGAGTTCCGCTGCCGGGATGGCA wild-type allele
AAGACGTGCTCC- - - - - - - - - - - CCGCTGCCGGGATGGCA mutant allele
AAGACGTGCTCCCC- - ACGAGTTCCGCTGCCGGGATGGCA mutant allele
BHK-21 Δ*ldlr-2* cells
CCAGGCCGCCACGTG- TGGGCCTGCCCAGTTCCGCTGCAAC wild-type allele
CCAGGCCGCCACGTG- - - GGCCTGCCCAGTTCCGCTGCAAC mutant allele
CCAGGCCGCCACGTGGTGGGCCTGCCCAGTTCCGCTGCAAC mutant allele
BHK-21 Δ*mxra8-1* cells
CGCGTGTACGAGCCGCGCGAC- - AACGGCCGCCTCCTGCTGT wild-type allele
CGCGTGTACGAGCCGC- CAAC- - - - - GGCCGCCTCCTGCTGT mutant allele
CGCGTGTACGAGCCGCGCGACACAACGGCCGCCTCCTGCTGT mutant allele
BHK-21 Δ*mxra8-2* cells
CCGACTCGTGGATATGTACT- CGGCGGGTGAACAGCGCGTG wild-type allele
CCGACTCGTGGATA- - - - - - - - - GCGGGTGAACAGCGCGTG mutant allele
CCGACTCGTGGATATGTACTTCGGCGGGTGAACAGCGCGTG mutant allele
BHK-21 Δ*ldlr*+Δ*mxra8* cells
Δ*ldlr:*
AAGACGTGCTCCCCGGA- - CGAGTTCCGCTGC- - - - CGGGATGGCA wild-type allele
AAGACGTGCTCCCCGGAAACGAGTTCCGCTGC- - - - CGGGATGGCA mutant allele
AAGACGTGCTCCCCGGAAACGAGTTCCGCTGCCGTGCGGGATGGCA mutant allele
Δ*mxra8:*
CCAGCGCGTGATCCACTGGGACCTCAGTGGTGTCCCTGGCAGCCAAGGGCGCCGACTCGTGGA
CCAGCGCGTGATCCACTGGGA- -
CCAGCGCGTGATCCACTGGGACCTCAGTGGTGTCCCTGGCAGCCAAGGGCGCCGACTCGTGGA

TATGTACTCGGCGGGTGAACAGCGCGTGTACGAGCCGCGCGACAACGGCCGCCTCCTGCTGTC
- -
TATGTACTCGGCGGGTGAACAGCGCGTGTACG - - - - - - - - - - - - - - - - CCGCCTCCTGCTGTC

GCCTTCTGCCTTCCAA wild-type allele
- CCTTCTGCCTTCCAA mutant allele
GCCTTCTGCCTTCCAA mutant allele

b

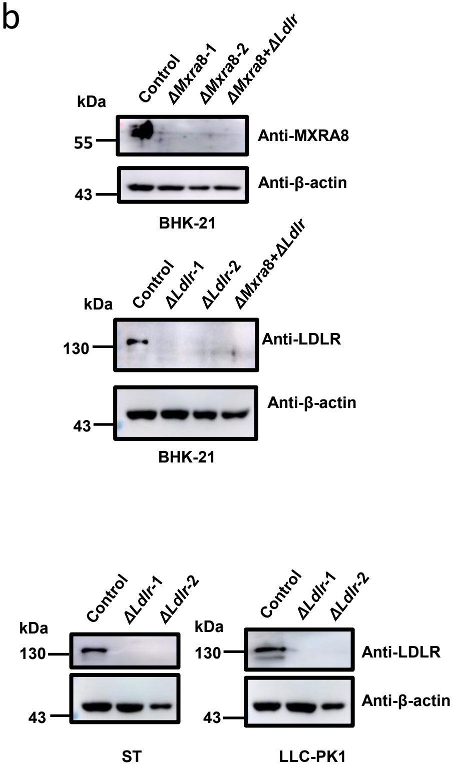

c

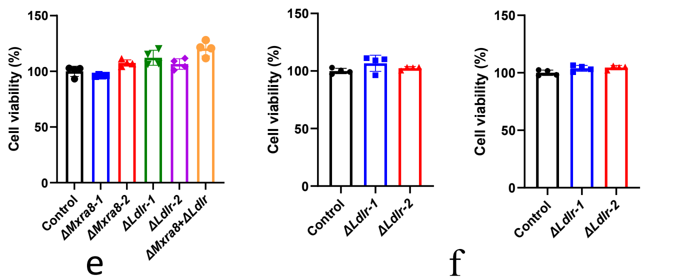

d

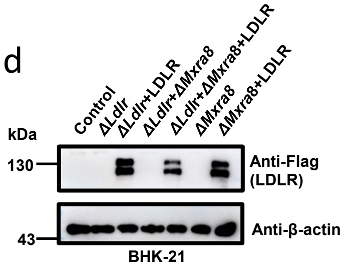

e

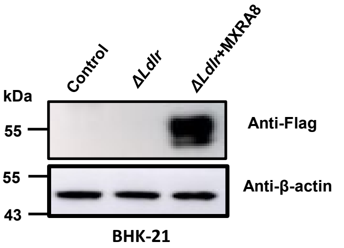

f

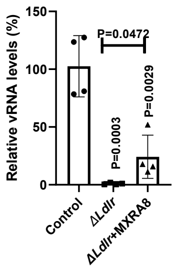

Supplementary Fig. 7 Knock-out of LDLR and/or MXRA8 expression by CRISPR–Cas9 gene editing. **a**, BHK-21, ST and LLC-PK1 cells were edited using a control or two different sgRNAs targeting *Ldlr* or *Mxra8*. Sanger sequencing was used to confirm successful *Ldlr* or *Mxra8* gene editing in obtained Δ*Mxra8* BHK-21, Δ*Ldlr* BHK-21, Δ*Mxra8*+Δ*Ldlr* BHK-21, Δ*LDLR* ST and Δ*LDLR* LLC-PK1 cell lines. **b**, western blot of lysates of parental (control) and *ΔMxra8* or *ΔLdlr* BHK-21, ST or LLC-PK1 cells. Expression on MXRA8 was detected using mouse polyclonal antibody and expression of LDLR was detected using rabbit monoclonal antibody. Antibody against beta-actin was used to detect the loading control. Representative images from two experiments are shown. **c**, Viability of parental BHK-21, LLC-PK1 and ST cells was compared to that of *Ldlr* or *Mxra8* gene knock-out cell lines by use of Cell Counting Kit 8. Data are mean ± SD of four biological replicates. **d**, The Δ*Mxra8*, Δ*Ldlr* or Δ*Mxra8*+Δ*Ldlr* BHK-21 cells were transiently transfected with expression plasmid for LDLR-Flag and expression of the protein was analyzed by western blot using monoclonal anti-Flag M2 antibody. Antibody against beta-actin was used to detect the loading control. **e**, **f**, Δ*Ldlr* BHK-21 cells were transfected with expression plasmid of MXRA8 or were mock-transfected. Cells were then infected with rGETV-EGFP (MOI=0.001). At 18 h post infection, copy numbers of virus genomes inside the cells were determined by qRT-PCR. Expression of tagged MXRA8 was verified using western blot analysis. Results are normalized relative to WT BHK-21 cells. Two-tailed p-values are calculated by unpaired Student's t test. The experiment was performed twice with similar results. Molecular masses are indicated in kDa (b, d, **e**). Source data are provided as a Source Data file.

Supplementary Fig. 8

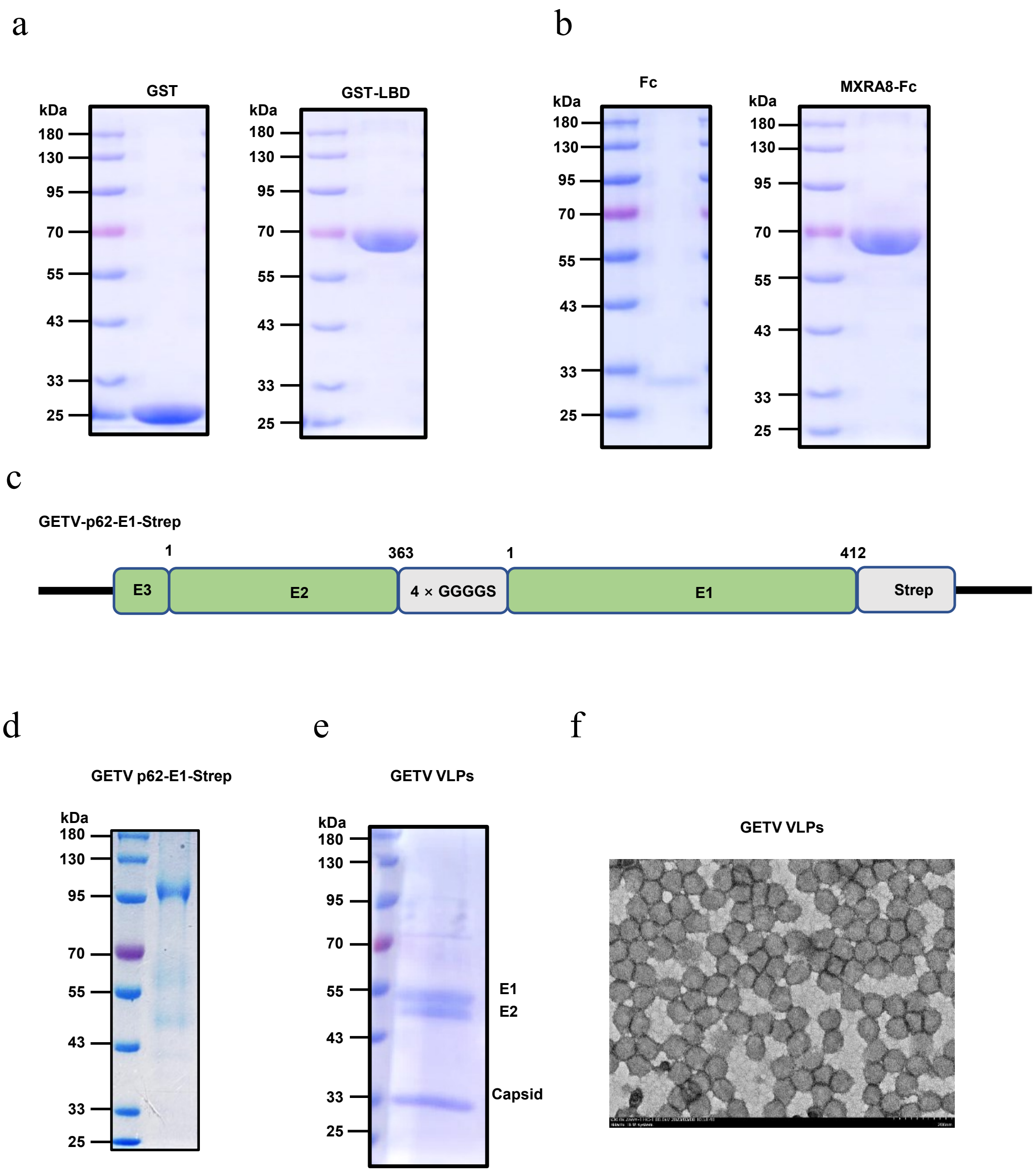

Supplementary Fig. 8 Analysis of purified recombinant proteins and VLPs preparations. a-b, Purified GST or GST-LBD fusion proteins (**a**), Fc or MXRA8-Fc fusion proteins (**b**) were separated using SDS-PAGE and detected by Coomassie blue staining. **c**, Schematic presentation of WT GETV structural polyprotein and expressed GETV p62-E1-Strep protein. TM, transmembrane domain; Strep, streptavidin binding tag. **d,** Purified GETV-p62-E1-strep polyprotein analyzed using SDS-PAGE and detected by Coomassie blue staining. **e**, Proteins present in purified VLPs of GETV were separated using SDS-PAGE and detected by Coomassie blue staining. **f**, Electron micrographs of negatively stained purified VLPs of GETV. Scale bar is 200 nm. The experiment was performed twice, and representative micrographs are shown. The experiment was performed twice with similar results. Molecular masses are indicated in kDa (**a-b, d-e**). Source data are provided as a Source Data file.

Supplementary Fig. 9

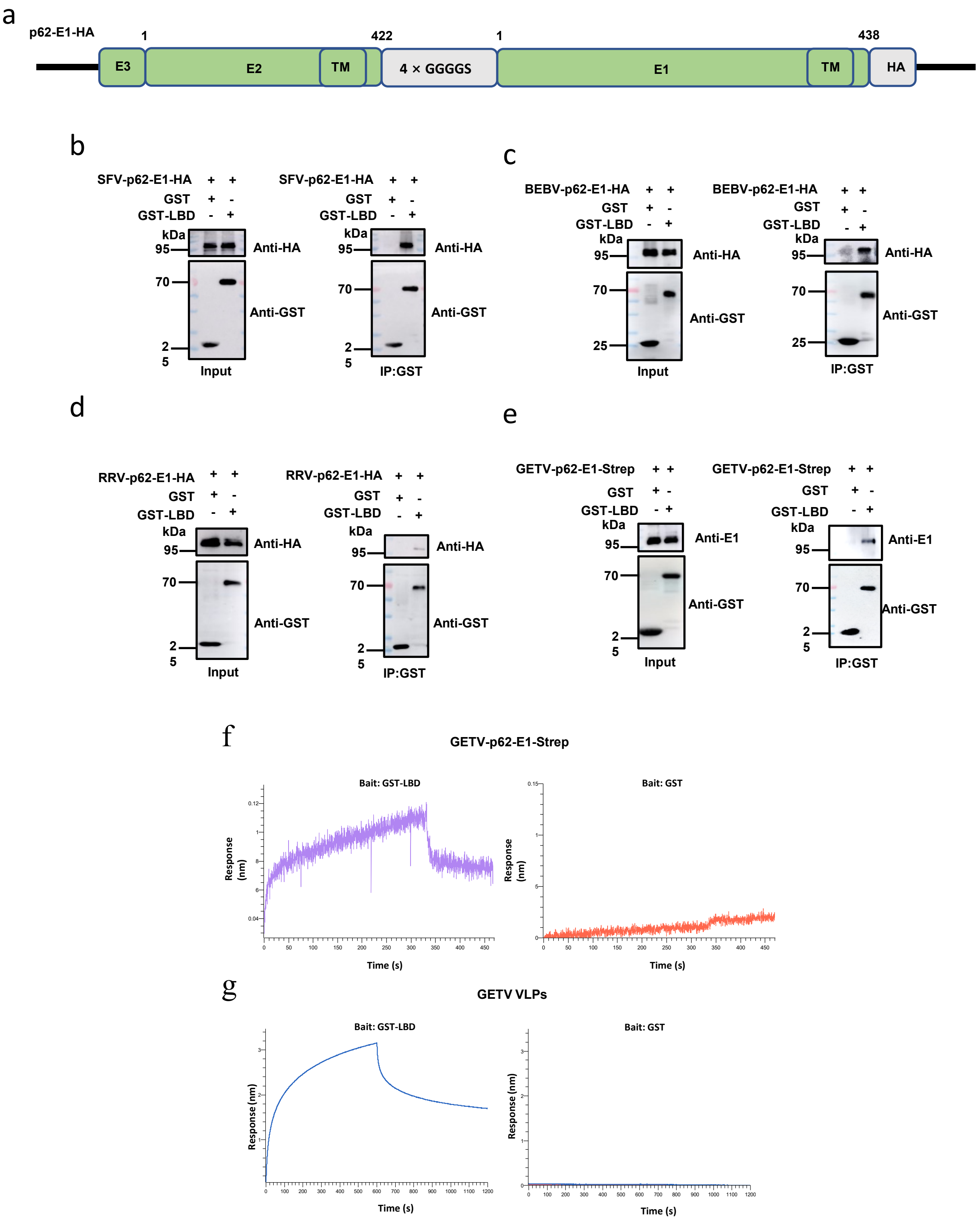

Supplementary Fig. 9 Demonstration of interaction between alphavirus E proteins or VLPs and GST-LBD protein. a, Scheme of the construct of p62-E1-HA plasmid. TM: transmembrane region. **b-d**, HA-tagged p62-E1 of SFV(**b**), BEBV(**c**) or RRV(**d**) was incubated with GST or GST-LBD and subsequently subjected to immunoprecipitation with anti-GST antibody. Proteins were detected using western blot analysis performed using antibodies against GST and HA tag. **e**, Purified GETV p62-E1-strep was incubated with GST or GST-LBD and subsequently subjected to immunoprecipitation with anti-GST antibody. Proteins were detected using western blot analysis performed using antibodies against GST and E1. **f**, Representative images of biolayer interferometry binding assay with the binding and disassociation parameters of GST-LBD and GST to GETV-p62-E1-strep. A 1:1 binding model was used to fit the experimental curves. **g**, Representative images of biolayer interferometry binding assay with the binding and disassociation parameters of GST-LBD and GST to GETV VLPs. A 1:1 binding model was used to fit the experimental curves. The experiment was performed twice with similar results. Molecular masses are indicated in kDa (**b-e**). Source data are provided as a Source Data file.

Supplementary Fig. 10

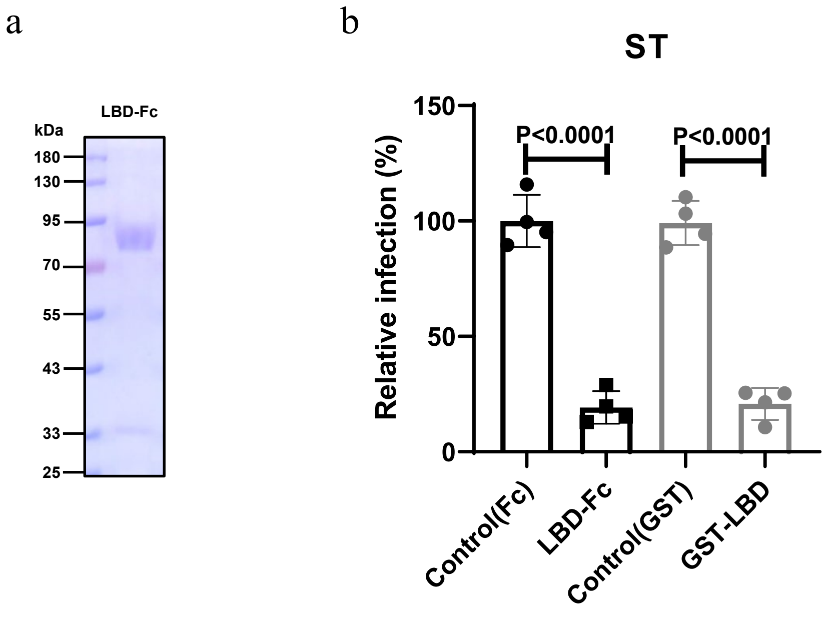

Supplementary Fig. 10 The LBD-Fc or GST-LBD fusion proteins block GETV infection. a, Purified LBD-Fc fusion protein analyzed using SDS-PAGE and detected by Coomassie blue staining. b, Virions of rGETV-EGFP were pre-incubated with LBD-Fc, Fc, GST-LBD or GST proteins taken at concentration 1 μg ml^{-1} and used to infect ST cells. At 18 h post infection number of infected cells was measured by flow cytometry. Results are normalized relative to control. Mean ± SD of two independent experiments. Two-tailed p-values are calculated by unpaired Student's t test. The experiment was performed twice with similar results. Molecular masses are indicated in kDa (**a**). Source data are provided as a Source Data file.

Supplementary Fig. 11

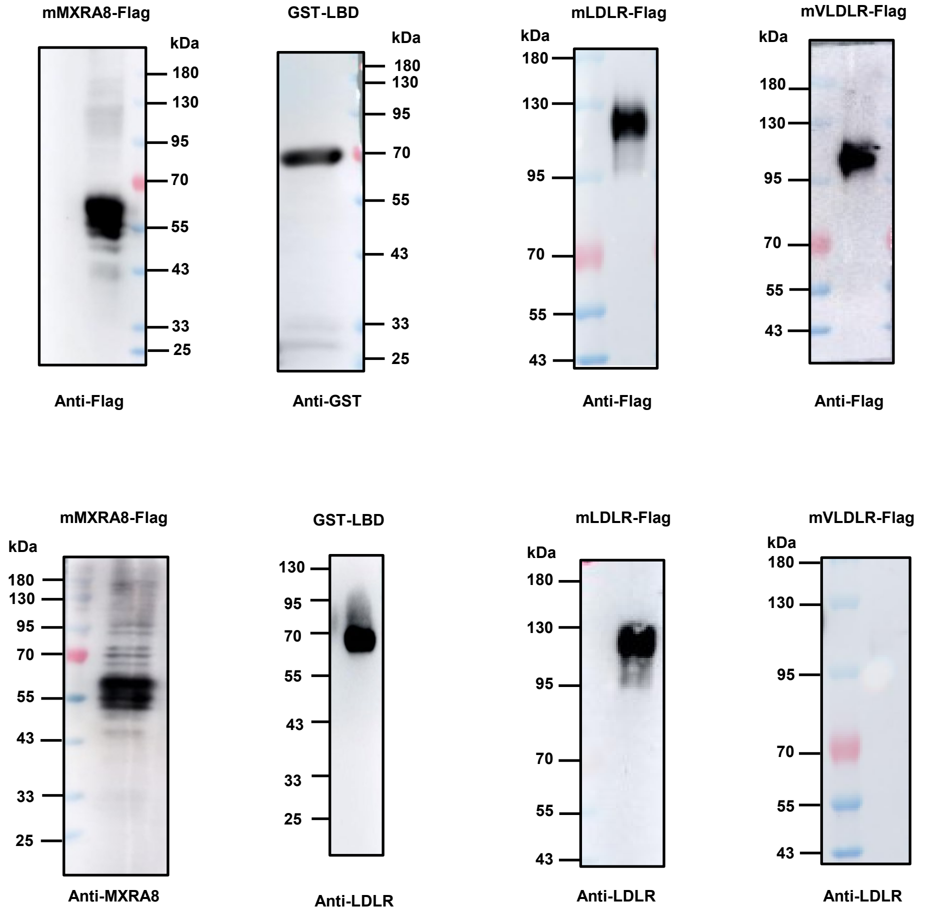

Supplementary Fig. 11 Antibody validation. Recombinant mMXRA8-Flag, GST-LBD, mLDLR-Flag and mVLDLR-Flag analyzed by western blot performed using antibodies to Flag-tag, GST, LDLR or MXRA8 as primary antibodies. The experiment was performed twice with similar results. Molecular masses are indicated in kDa. Source data are provided as a Source Data file.

Supplementary Fig. 12

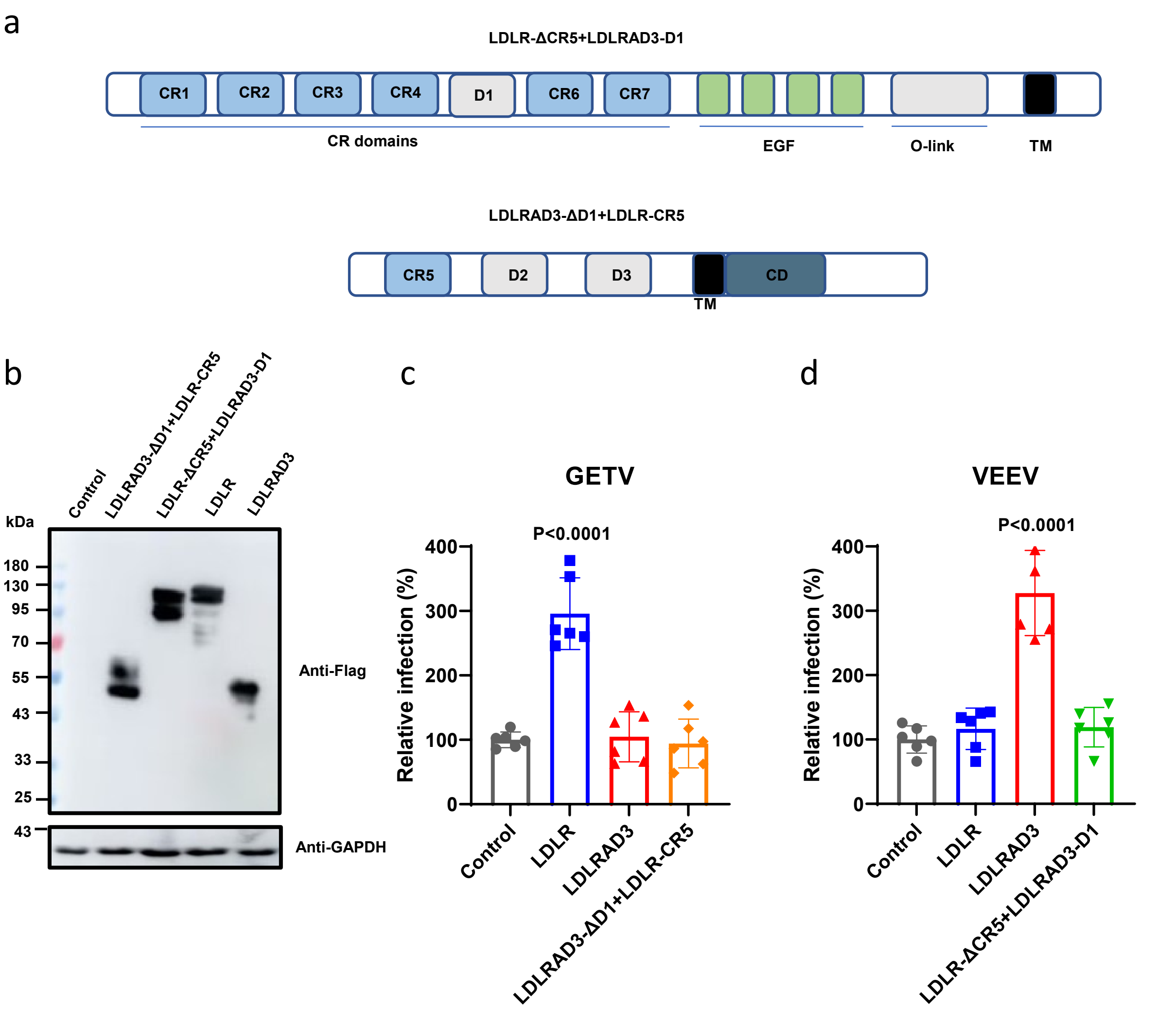

Supplementary Fig. 12 LDLR and LDLRAD3 chimeric proteins do not promoter infection with pseudo-virions GETV or VEEV. a, A schematic diagram describing how the LDLR-deltaCR5+LDLRAD3-D1 construct was designed. CD: cytoplasmic domain; TM: transmembrane region. **b,** HEK 293T cells were transiently transfected with plasmids encoding LDLRAD3-ΔD1+LDLR-CR5, LDLR-ΔCR5+LDLRAD3-D1, LDLRAD3 or LDLR. Expression of recombinant proteins was detected by western blot analysis using monoclonal anti-Flag M2 antibody, anti-GAPDH antibody was used to detect loading control. **c**, **d**, The efficiency of infection of HEK-293T cells, transiently transfected with expression constructs of indicated proteins, using GETV and VEEV pseudo-viruses was measured by detection of EGFP positive cells by flow cytometry. Results are normalized relative to control. Mean ± SD of three independent experiments performed in duplicates are shown. Two-tailed p-values are calculated by unpaired Student's t test. The experiment was performed twice with similar results. Molecular masses are indicated in kDa (**b**). Source data are provided as a Source Data file.

Supplementary Fig. 13

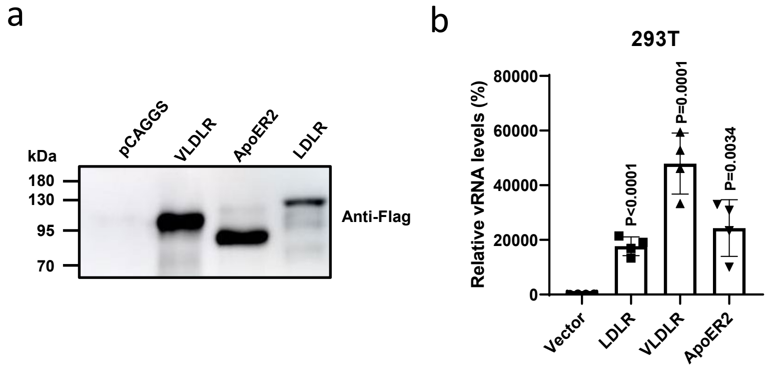

Supplementary Fig. 13 The effects of LDLR, VLDLR or ApoER2 on GETV. a, HEK 293T cells were transfected with empty pCAGGS vector or with expression plasmids for hamster LDLR, VLDLR, and ApoER2. western blot was made using anti-Flag antibody. **b**, HEK 293T cells expressing hamster LDLR, VLDLR or ApoER2 or transfected with pCAGGS vector (negative control) were infected with the GETV-HN. At 12 h post infection viral RNA (vRNA) levels in infected cells were measured via qRT-PCR. vRNA levels in control cells were taken as 100%. . Mean ± SD of two independent experiments. Two-tailed p-values are calculated by unpaired Student's t test. The experiment was performed twice with similar results. Molecular masses are indicated in kDa (**a**). Source data are provided as a Source Data file.

Supplementary Fig. 14

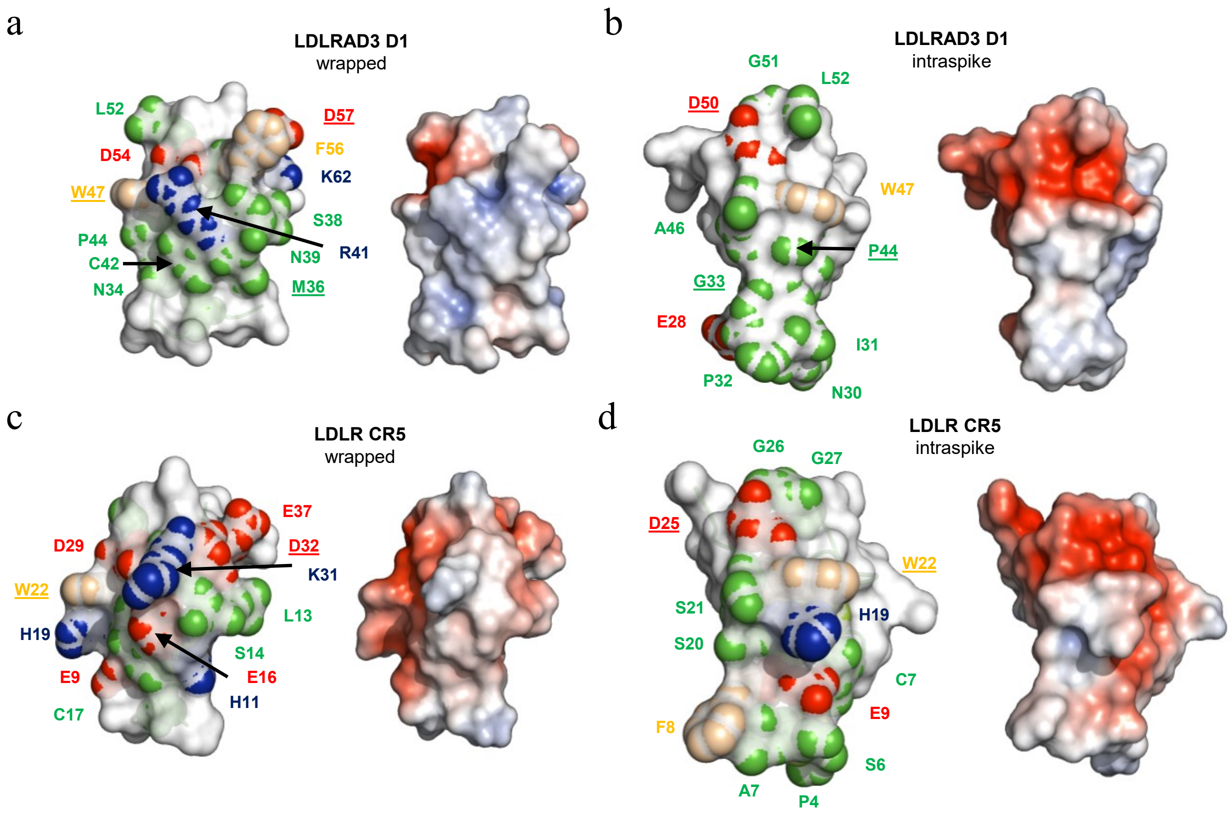

Supplementary Fig. 14 Comparison of the molecular surfaces of D1 of LDLRAD3 and CR5 of LDLR. a-b, Surface representation of D1 of LDLRAD3 showing the amino acid involved in binding to E1/E2 of VEEV as spheres. **a**, Wrapped form, donates contacts to the wrapped E2-E1 heterodimer **b**, Intraspike form, intraspike refers to the intraspike E2-E1 heterodimer. **c-d,** Surface representation of CR5 of LDLR showing the amino acid located at equivalent positions as residues shown in **a** and **b** that important for binding of D1 of LDLRAD3 to E1-E2 of VEEV. Positively charged residues are coloured blue, negatively charged red, aromatic residues in wheat and other amino acids in green. The right part of each figure shows the electrostatic surface potential of the same view of the structure, color-coded from red (acidic) to blue (basic). The deep red in the upper part of both structures corresponds to the acidic residues that coordinate the calcium ion.

Supplementary Fig. 15

a

```
Clustal Omega CR4 alignment
Human    CR4      TCGPAS-FQCNSSTCIPQLWACDNDPDCEDGSDEWPQRCRG
Mouse    CR4      TCGPAH-FRCNSSICIPSLWACDGDVDCVDGSDEWPQNCQG
Rabit    CR4      TCGPAH-FRCNSSSCVPALWACDGEPDCDDGSDEWPARCGA
Horse    CR4      TCGPAS-FQCNSSACIPELWACDGDPDCKDGSDEWPQRCGA
Pig      CR4      TCGPAS-FQCNSSTCIPELWACDGDPDCEDGSDEWPQHCRS
Cattle   CR4      TCGPAN-FQCNSSMCIPQLWACDGDPDCDDGSDEWPKHCGT
Goat     CR4      TCGPAN-FQCNSSMCIPQLWACDDDPDCDDGSDEWPKHCGS
Mosquito CR4     TCDPLKQFACSENYCITSKWRCDGEPDCPDGSDERGCTNPT
                  *****  *:**** *:* *****.: ** ******* .*

Clustal Omega CR5 alignment
Human    CR5      SSPCSAFEFHCLS-GECIHSSWRCDGGPDCKDKSDEENCAV
Mouse    CR5      SSPCSSLEFHCGS-SECIHRSWVCDGEADCKDKSDE-ECAV
Rabit    CR5      RGPCSRHEFHCGS-GECVHASWRCDGDADCRDGSDERDCAA
Horse    CR5      DSPCSALEFHCSS-GECVHSSWRCDGDPDCRDKSDEDNCAV
Pig      CR5      NNPCSALEFHCHS-GECIHSSWRCDGDTDCKDKSDEENCDV
Cattle   CR5      NNPCSALEFHCGS-GECIHSSWHCDHDPDCKDKSDEENCAV
Goat     CR5      DNPCSALEFHCGS-GECIHSSWRCDRDPDCKDKSDEENCAV
Mosquito CR5     VNPCLSLEYQCSDRITCIHKSWICDGEKDCPDGDDEMPPIC
                   .***  **** * .**:* ** **   **:* *** :*  .
```

b

RMSD = 1.291 (30 to 30 atoms)

```
Cr2 CKSGDFSCGGRVNRCIPQFWRCDGQVDCDNGSDEQGC
    |.:.:|.|  ....||...|||||..||.:.|||:.|
Cr5 CSAFEFHC--LSGECIHSSWRCDGGPDCKDKSDEENC
```

RMSD = 1.362 (32 atoms)

```
Cr5 CSAFEFHCLSGECIHSSWRCDGGPDCKDKSDEENC
    |...||.|..|.|||.|.:||...||||.|||..|
Cr6 CRPDEFQCSDGNCIHGSRQCDREYDCKDLSDEVGC
```

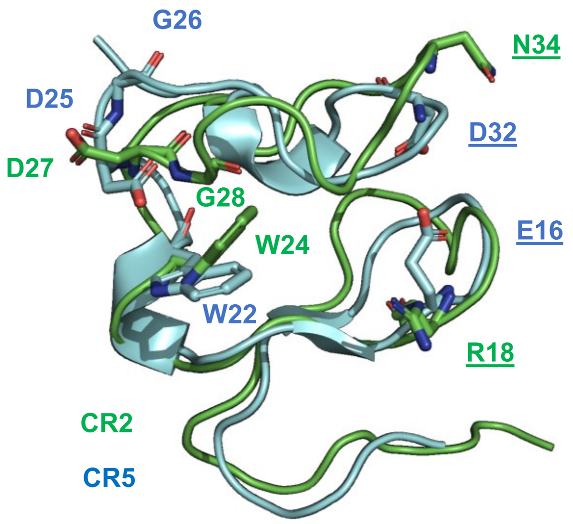

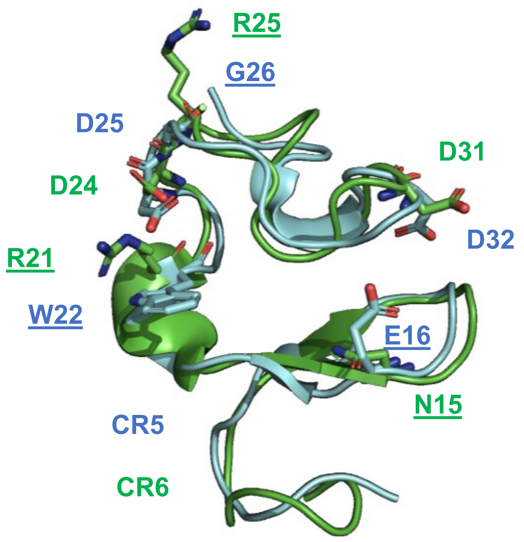

Supplementary Fig. 15 Conservation of amino acids important for binding of GETV to the CR5 domain. a, Alignment of sequences of the CR4 and CR5 domains of LDLR of human, mouse, mosquito and farm animals susceptible to GETV infection. The amino acids sequences of the CR4 and CR5 domains of the LDLR receptor from human (NP_000518.1), mouse (NP_034830.2), rabbit (XP_051693990.1), horse (XP_005611994.2), pig (NP_001193283.1), cattle (NP_001160002.1), goat (XP_005682432.1) and Mosquito (AAQ16410.1) were aligned using Clustal Omega server (https://www.ebi.ac.uk/Tools/msa/clustalo/). Amino acids residues, replacement of which led to the strongest reduction of ability to promote the cell entry of GETV, are in red, those with a lower impact are in blue and these for which no detectable effect was revealed are in bold. The amino acids residues coordinating the binding of Ca^{2+}-ion are highlighted in grey. The six cysteine residues forming three disulfide-linkages are also completely conserved. **b**, Conservation of amino acids important for binding of GETV to the CR5 domain in CR2 and CR6. The 3D-structures of CR5 (PDB: 1AJJ) and CR2 (1LDR) and CR6 (1D2J) were aligned and the amino acids important in CR5 for binding to GETV or the corresponding amino acids in CR2 and CR6 are highlighted as sticks. The two and three non-conservative substitutions are underlined. The upper part shows a pairwise alignment of the sequences using emboss water (https://www.ebi.ac.uk/Tools/psa/emboss_water/)

Supplementary Table 1 Membrane protein library information

	Gene	Red fluorescence intensity	Green fluorescence intensity
1	ANXA7	47	1341
2	BCL2L13	77	4891
3	CXCL9	42	832
4	FNDC5	42	502
5	CD38	41	654
6	HMOX1	44	654
7	IGF1R	19	682
8	PPP3CB	30	675
9	SLC1A2	49	1571
10	RAB35	18	658
11	ASGR1	34	541
12	CDC42	51	673
13	CAMK2A	54	327
14	FAS	29	313
15	NFE212	32	381
16	HSPA5	56	735
17	IFNGR1	23	1413
18	PTEN	34	527
19	SLC12A5	30	587
20	LDLR	351	662
21	ACSL1	67	752
22	CLEC1A	44	327
23	CYLD	44	3455
24	FOLH1	24	1323
25	GIT1	37	586
26	HCK	41	1955
27	JAG1	21	658
28	P2RX2	19	1039
29	SCAMP5	28	819
30	TIMD4	244	641
31	ADSSL1	75	2999
32	CRTC1	41	926
33	CHD1	26	668
34	FGFR4	16	620
35	PDCD1	28	671
36	HSP90B1	14	1300
37	LRP3	10	590
38	P2RY14	8	515
39	SERPINE2	21	507
40	APOE	47	528
41	CACNA2D2	40	350
42	DLL1	9	338
43	FBXW7	26	559
44	GNAI3	23	643
45	HPSE	46	274
46	LAMP2	11	640
47	PITPNA	55	588
48	SIGMAR	1	661
49	ATG14	45	775
50	CTNNB1	41	420
51	EPHB2	6	373
52	CNTNAP4	25	302
53	HCN2	15	393
54	GORASP1	34	390
55	LGALS3BP	21	405
56	PML	40	1068
57	TH	24	855
58	AKAP5	52	921
59	CXADR	33	520
60	EFR3A	32	407
61	SIRT2	27	586
62	AGER	47	559
63	SREBF2	34	350
64	PTPN6	43	562
65	P4HB	39	1356
66	TRPA1	30	791
67	ARC	78	594
68	CTNND1	27	888
69	EPHB4	20	614
70	GSDMD	56	1282
71	PHB2	56	656
72	HOMER1	38	701
73	PIK3R3	10	773
74	SOX10	3	994
75	TNFRSF1A	37	778
76	ARRB2	36	752
77	CD36	47	284
78	CD209D	42	278
79	ICOSL	48	281
80	MAS1	42	243
81	NUMB	35	324
82	RAB7	42	310
83	SOCS1	43	361
84	SLC40A1	23	333
85	XBP1	61	383
86	APLNR	32	365
87	CCR8	32	248
88	DLG2	32	274
89	IFNG	41	464
90	MAPK1	24	313
91	OLFR168	32	366
92	RAB5B	43	317
93	SOCS3	38	519
94	TM4SF19	38	526
95	ZHX2	42	385
96	ACKR4	24	421
97	CX3CR1	21	283
98	FAP	25	307
99	ITGB1	11	401
100	MRGPRD	1	265
101	OSCP1	15	706
102	RNF217	12	222
103	SCARB1	20	296
104	TSPO	11	1619
105	APOO	27	323
106	CD209B	24	409
107	GJB1	24	578
108	ITGA4	6	306
109	MTDH	12	293
110	ORAI1	4	375
111	SDC1	20	263
112	SPINK5	31	2347
113	TLR8	14	256
114	ANXA5	41	385
115	CLSTN1	30	448
116	GRM5	16	320
117	KCNJ11	14	343
118	MAPK3	12	460
119	PTGS2	7	265
120	SEMA3E	9	277
121	SLC19A2	12	269
122	TNFRSF4	8	309
123	AP4M1	62	557
124	CHL1	25	552
125	GRIN2A	5	428
126	KCNN4	9	266
127	MYSM1	7	272
128	PCSK9	24	298
129	SMURF1	9	287
130	SPARC	24	374
131	VTCN1	7	320
132	BCL3	10	297
133	CD200	23	400
134	GPBAR1	12	261
135	LGR4	17	278
136	NCAM1	16	293
137	PRKCG	26	331
138	SHH	35	322
139	SLC17A8	21	321
140	VDAC1	7	322
141	CD47	59	414
142	CXCR4	55	389
143	HPCAL1	25	2336
144	LGALS3	15	1910
145	NOS1AP	6	418
146	PLG	31	272
147	SYT1	42	598
148	SLC6A6	51	301
149	WNT7B	26	347
150	LDLRAD3	43	1090

Supplementary Table 2 ELISA reactivity of mouse anti-LDLR or anti-MXRA8 polyclonal antibodies (pAbs) against GST-LBD or MXRA8-Fc

Serum dilution ratio	Isotype	Anti-LDLR	Anti-MXRA8
10^{-1}	0.2316	3.0249	3.247
10^{-2}	0.3671	2.9713	2.783
10^{-3}	0.0725	2.9537	2.323
10^{-4}	0.0516	2.8227	1.875
10^{-5}	0.0504	1.2647	1.423
10^{-6}	0.0497	0.2133	1.071
10^{-7}	0.0417	0.0641	0.891
10^{-8}	0.0629	0.0543	0.458

Supplementary Table 3 Receptors of Alphaviruses

Virus	LDLR	LDLRAD3	VLDLR	ApoER2	MXRA8
GETV	+	-	+	+	+
SFV	+	-	+	+	-
BEBV	+	-	n.a.	n.a.	+
RRV	+	-	n.a.	n.a.	+
VEEV	-	+	-	-	-
CHIKV	-	-	-	-	+
MAYV	-	-	n.a.	n.a.	+
MIDV	-	-	n.a.	n.a.	+

+: serves as entry factor, does not serve as entry factor, n.a.: not analysed.

Supplementary Table 4 Sequences of the primers used for PCR

Gene	Sequence (5′–3′)
Mes-*Ldlr*-sg-F1	caccGCCGGCAGCGGAACTCGTCCG
Mes-*Ldlr*-sg-R1	aaacCGGACGAGTTCCGCTGCCGGC
Mes-*Ldlr*-sg-F2	caccGGAACTGGGCAGGCCCACACG
Mes-*Ldlr*-sg-R2	aaacCGTGTGGGCCTGCCCAGTTCC
Mes-*Mxra8*-sg-F1	caccGGTGTACGAGCCGCGCGACAA
Mes-*Mxra8*-sg-R1	aaacTTGTCGCGCGGCTCGTACACC
Mes-Mxra8-sg-F2	caccG ACTCGTGGATATGTACTCGG
Mes-Mxra8-sg-R2	aaacCCGAGTACATATCCACGAGTC
Sus-*LDLR*-sg-F1	caccGTGATGGTGATCCTGACTGCG
Sus-*LDLR*-sg-R1	aaacCGCAGTCAGGATCACCATCAC
Sus-*LDLR*-sg-F2	caccGACAGCTCATCACTCCCCGAG
Sus-*LDLR*-sg-R2	aaacCTCGGGGAGTGATGAGCTGTC
pCDH-pLDLR-F	GATTCTAGAGCTAGCGAATTCATGAAGTCCACGGGCTGGG
pCDH-pLDLR-R	ATCCTTGTAATCTTCGAACGCCACGTCATCCTCCAA
pCDH-mes-LDLR-F	GATTCTAGAGCTAGCGAATTCATGCGCACCGCGGATCTA
pCDH-mes-LDLR-R	ATCCTTGTAATCTTCGAATGCCACATCATCCTCCAGGC
pCDH-mes-LDLRAD3-F	GATTCTAGAGCTAGCGAATTCATGTGGCTGCTAGGGCCG
pCDH-mes-LDLRAD3-R	ATCCTTGTAATCTTCGAATACTTCTTCAGTGCCCTCATTGG
pCDH-mes-MXRA8-F	GATTCTAGAGCTAGC GAATTC ATGGAACTGCTGTCCTGTGTCTT
pCDH-mes-MXRA8-R	ATCCTTGTAATCTTCGAA TTTGCAGTACTCCTTCCTGAACTCT
Pca-YH-pLDLR-F	TTTGGCAAAGAATTCATGAAGAGCACCGGGTGGG
Pca-YH-pLDLR-R	CTTGTAGTCGGTACCGGCCACGTCATCCTCCAGG
pCA-mes-LDLR-F	TTTGGCAAAGAATTCATGCGCACCGCGGATCTA
pCA-mes-LDLR-R	CTTGTAGTCGGTACCTGCCACATCATCCTCCAGGC
pCA-mes-LDLRAD3-F	TTTGGCAAAGAATTCATGTGGCTGCTAGGGCCG
pCA-mes-LDLRAD3-R	CTTGTAGTCGGTACCTACTTCTTCAGTGCCCTCATTGG
pCA-mes-MXRA8-F	TTTGGCAAAGAATTC ATGGAACTGCTGTCCTGTGTCTT
pCA-mes-MXRA8-R	CTTGTAGTCGGTACCTTTGCAGTACTCCTTCCTGAACTCT
qPCR-BEBV-E2-F	AACTCTCCTTTCGTCCCTAGA
qPCR-BEBV-E2-R	TTGCCGTGTTCCACGATAG
qPCR-SFV-E2-F	GAGATCCCTTGCACCACTTATC
qPCR-SFV-E2-R	CTTTCCTCCGACTGTGATCTTT
qPCR-RRV-E2-F	GTGCATGTTCCATTCCCTTTG
qPCR-RRV-E2-R	GTCGGATGATCTGGGTGTAATC
qPCR-GETV-E2-F	AGAGAACTGACGGTGAAACTG
qPCR-GETV-E2-R	CCATCTGTACTCGATCCCTTC
qPCR-human-GAPDH-F	GCACCGTCAAGGCTGAGAAC
qPCR-human-GAPDH-R	TGGTGAAGACGCCAGTGGA
qPCR-PRV-F	GTCACCCGCGTGCTGATCGTCT
qPCR-PRV-R	GGCAACCACCGGCGCTACTTT
qPCR-SVV-F	GCTCCTGGCTCTAACCATACTAAT
qPCR-SVV-R	GACAAAAGTAACCATCGTCCTGCT
qPCR-hamster-β-actin-F	GGTGG GAATGGGTCAGAAG
qPCR-hamster-β-actin-R	AGCTCATTGTAGAAGGTG TGG
