# Supplementary material for: LDLR is used as a cell entry receptor by multiple alphaviruses
Source: Nat Commun. 2024 Jan 20;15:622. doi: 10.1038/s41467-024-44872-5 (PMC10799924; doi:10.1038/s41467-024-44872-5)
Supplement: Supplementary file 3 — Reporting Summary [file 41467_2024_44872_MOESM3_ESM.pdf]

nature portfolio

Corresponding author(s): Shuo Su

Last updated by author(s): Dec 15, 2023

Reporting Summary

Nature Portfolio wishes to improve the reproducibility of the work that we publish. This form provides structure for consistency and transparency in reporting. For further information on Nature Portfolio policies, see our Editorial Policies and the Editorial Policy Checklist.

Statistics

For all statistical analyses, confirm that the following items are present in the figure legend, table legend, main text, or Methods section.

n/a	Confirmed	
☐	☒	The exact sample size (*n*) for each experimental group/condition, given as a discrete number and unit of measurement
☐	☒	A statement on whether measurements were taken from distinct samples or whether the same sample was measured repeatedly
☐	☒	The statistical test(s) used AND whether they are one- or two-sided<br>*Only common tests should be described solely by name; describe more complex techniques in the Methods section.*
☐	☒	A description of all covariates tested
☐	☒	A description of any assumptions or corrections, such as tests of normality and adjustment for multiple comparisons
☐	☒	A full description of the statistical parameters including central tendency (e.g. means) or other basic estimates (e.g. regression coefficient) AND variation (e.g. standard deviation) or associated estimates of uncertainty (e.g. confidence intervals)
☐	☒	For null hypothesis testing, the test statistic (e.g. *F*, *t*, *r*) with confidence intervals, effect sizes, degrees of freedom and *P* value noted<br>*Give P values as exact values whenever suitable.*
☒	☐	For Bayesian analysis, information on the choice of priors and Markov chain Monte Carlo settings
☒	☐	For hierarchical and complex designs, identification of the appropriate level for tests and full reporting of outcomes
☒	☐	Estimates of effect sizes (e.g. Cohen's *d*, Pearson's *r*), indicating how they were calculated

Our web collection on statistics for biologists contains articles on many of the points above.

Software and code

Policy information about availability of computer code

Data collection	The OD450 value is obtained by Tecan i-control Software on TECAN Infinite M200 Pro; Flow cytometry data were acquired by Accuri CFlow Software on flow cytometry (BD Accuri® C6); Biolayer interferometry (BLI) binding assay were performed using Data Acquisition Software on Octet RED96 Instrument(Pall Fortebio); Data of qRT-PCR experiments are collected in LightCycler@ 96 Instrument Software; Amersham Imager 600 was used to collect the western blot figures, Nikon A1 confocal microscope (Japan) are used to collect the fluorescence images.
Data analysis	FlowJo version 10, MAGeCK,GraphPad Prism 9, Data Analysis version 12.0 software (ForteBio), NIS-Elements AR 4.51.00 (Nikon, Japan).

For manuscripts utilizing custom algorithms or software that are central to the research but not yet described in published literature, software must be made available to editors and reviewers. We strongly encourage code deposition in a community repository (e.g. GitHub). See the Nature Portfolio guidelines for submitting code & software for further information.

Data

Policy information about availability of data

All manuscripts must include a data availability statement. This statement should provide the following information, where applicable:

- Accession codes, unique identifiers, or web links for publicly available datasets
- A description of any restrictions on data availability
- For clinical datasets or third party data, please ensure that the statement adheres to our policy

The authors declare that all data supporting the findings of this study are available within the paper and its Supplementary information.

Research involving human participants, their data, or biological material

Policy information about studies with human participants or human data. See also policy information about sex, gender (identity/presentation), and sexual orientation and race, ethnicity and racism.

Reporting on sex and gender	No human data
Reporting on race, ethnicity, or other socially relevant groupings	No human data
Population characteristics	No human data
Recruitment	No human data
Ethics oversight	No human data

Note that full information on the approval of the study protocol must also be provided in the manuscript.

Field-specific reporting

Please select the one below that is the best fit for your research. If you are not sure, read the appropriate sections before making your selection.

- [x] Life sciences
- [ ] Behavioural & social sciences
- [ ] Ecological, evolutionary & environmental sciences

For a reference copy of the document with all sections, see nature.com/documents/nr-reporting-summary-flat.pdf

Life sciences study design

All studies must disclose on these points even when the disclosure is negative.

Sample size	The number of cells in Figure 2b is automatically set by ImageJ. Sample sizes for mouse studies were determined based on previous results for similar in vivo experiments (PMID: 34986000, PMID: 34929721).No sample-size calculations were performed to power each study.
Data exclusions	No data was excluded.
Replication	All experiments had at least 2 independent biological replicates. All replication attempts were successful.
Randomization	For animal studies, mice were randomly assigned to treatment groups. For in vitro studies, sample allocation was not randomized because the results are quantitative and did not require subjective judgment or interpretation. This practice is standard in the field (PMID: 34929721).
Blinding	Not blinded. The key experiments were repeated independently by multiple members of the laboratory. Blinding was not deemed necessary because the results are quantitative and did not require subjective judgment or interpretation. Blinding is also not typically used in the field for similar in vitro and in vivo studies (PMID: 34929721).

Reporting for specific materials, systems and methods

We require information from authors about some types of materials, experimental systems and methods used in many studies. Here, indicate whether each material, system or method listed is relevant to your study. If you are not sure if a list item applies to your research, read the appropriate section before selecting a response.

Materials & experimental systems

n/a	Involved in the study	
[ ]	[x]	Antibodies
[ ]	[x]	Eukaryotic cell lines
[x]	[ ]	Palaeontology and archaeology
[ ]	[x]	Animals and other organisms
[x]	[ ]	Clinical data
[x]	[ ]	Dual use research of concern
[x]	[ ]	Plants

Methods

n/a	Involved in the study	
[x]	[ ]	ChIP-seq
[ ]	[x]	Flow cytometry
[x]	[ ]	MRI-based neuroimaging

Antibodies

Antibodies used	FITC conjugated goat anti-mouse IgG Fc (Thermo Fisher, 31547), polyclonal anti-LDLR mouse antiserum,LDL Receptor (LDLR) rabbit mAb (ABclonal, A20808), GST tag polyclonal antibody (Proteintech, 10000-0-AP), mCherry-Tag (4C16) mouse mAb (Abmart M40012),

	monoclonal anti-Flag M2 antibody (Sigma, F1804), monoclonal anti-HA antibody (Sigma, H3663), beta actin monoclonal antibody (Proteintech, 66009), anti-MXRA8, GETV 3H2 anti-E1, 4D10 anti-E1, 8D5 anti-E2 monoclonal antibodies, and GETV anti-capsid protein polyclonal antibody (in-house).
Validation	1. FITC conjugated goat anti-mouse IgG Fc (Thermo Fisher, 31547); Commercially validated by IF, ICC, IHC, IP and FACS.<br>2.Polyclonal anti-LDLR mouse antiserum generated in our laboratories, (Validated by SDS-PAGE analysis and binding to GST-LBD recombinant proteins); Yu Wang and xiaofeng zhai, unpublished.<br>3.LDL Receptor (LDLR) rabbit mAb (ABclonal, A20808); Commercially validated by Western blot and immunofluorescence.<br>4.GST tag polyclonal antibody (Proteintech, 10000-0-AP); Commercially validated by Western blot and flow cytometry.<br>5. mCherry-Tag (4C16) mouse mAb (Abmart M40012); Commercially validated by Western blot.<br>6.Monoclonal anti-Flag M2 antibody (Sigma, F1804); Commercially validated by Western blot,immunoprecipitation, immunofluorescence and flow cytometry.<br>7.Monoclonal anti-HA antibody (Sigma, H3663); Commercially validated by Western blot, immunocytochemistry and immunoprecipitation .<br>8.beta actin monoclonal antibody (Proteintech, 66009);Commercially validated by Western blot.<br>9.Anti-MXRA8 polyclonal mouse serum generated in our laboratories, (Validated by SDS-PAGE analysis and binding MXRA8-Flag proteins); Yu Wang and xiaofeng zhai, unpublished.<br>10.GETV 3H2 anti-E1, 4D10 anti-E1, 8D5 anti-E2 monoclonal antibodies, and GETV anti-capsid protein polyclonal antibody generated in our laboratories, (Validated by SDS-PAGE analysis and binding to GETV-infected cells); Yu Wang and xiaofeng zhai, unpublished.

Eukaryotic cell lines

Policy information about cell lines and Sex and Gender in Research

Cell line source(s)	HEK 293T (ATCC CRL-3216), BHK-21 (ATCC CCL-10), LLC-PK1 (ATCC CL-101), ST (ATCC CRL-1746) and Vero cells (ATCC CCL-81) were obtained from ATCC. Expi293F cells (Thermo Fisher, A14635)were obtained from Thermo Fisher.
Authentication	Commercially validated cell lines were obtained from ATCC and other sources and grew and performed as expected. Morphology of each cell line was assessed by microscopy.
Mycoplasma contamination	All cell lines are routinely tested each month and were negative for mycoplasma.
Commonly misidentified lines<br>(See ICLAC register)	This study did not involve any commonly misidentified cell lines.

Animals and other research organisms

Policy information about studies involving animals; ARRIVE guidelines recommended for reporting animal research, and Sex and Gender in Research

Laboratory animals	The two-day-old ICR mice, 6-weeks old ICR mice and 6-week-old BALB/c mice were used. Pups were taken as mixed groups and were not sexed (e.g., both sexes were used). Mice were fed a 19% protein diet (Harlan Teklad, Irradiated), had 12 h light/dark cycle (0600-1800), and were housed in a facility maintained at a temperature range of 20-26 °C with a humidity range of 30–70.
Wild animals	No wild animals were involved in the study.
Reporting on sex	Both sexes were used.
Field-collected samples	No field-collected samples were used in this study.
Ethics oversight	The animal experiments were approved by the Institutional Animal Care and Use Committee of Nanjing Agricultural University, Nanjing, China (permission SYXK2017-0007; February 2017).

Note that full information on the approval of the study protocol must also be provided in the manuscript.

Flow Cytometry

Plots

Confirm that:

- [x] The axis labels state the marker and fluorochrome used (e.g. CD4-FITC).
- [x] The axis scales are clearly visible. Include numbers along axes only for bottom left plot of group (a 'group' is an analysis of identical markers).
- [x] All plots are contour plots with outliers or pseudocolor plots.
- [x] A numerical value for number of cells or percentage (with statistics) is provided.

Methodology

Sample preparation	Cells expressing marker with green fluorescence were trypsinized and resuspended with PBS at the selected time points, and quantified by flow cytometry. For staining of cells that express Flag-tagged proteins or for counting of cells infected with GETV-HN, GETV-GX, and GETV-FJ, which do not express any fluorescence, adhered cells were fixed with 4% (v/v) paraformaldehyde (PFA) for 30 min, and then permeabilized with 0.1% Triton X-100 in PBS for 20 min. Cells were incubated with appropriate specific antibody indicated in the figure legends for 2 h at room temperature. After washing three times with 0.01 % Triton X-100 in PBS, cells were incubated with Alexa Fluor 488 conjugated goat anti-mouse IgG diluted in 0.01% Triton X-100 in PBS for 30 min at room temperature and then the cells positive for selected protein expression were analyzed by flow cytometry.
Instrument	flow cytometry (BD Accuri® C6)
Software	FlowJo v.10(BD)
Cell population abundance	For sorted cell stable lines, purity was confirmed by cell surface staining after expansion in media containing puromycin.
Gating strategy	Gating was performed based on non-binding control antibodies and/or uninfected cells. Dead cells and cell debris were excluded by scatter and size.

☒ Tick this box to confirm that a figure exemplifying the gating strategy is provided in the Supplementary Information.
